# Group versus individual sessions delivered by a physiotherapist for female urinary incontinence: an interview study with women attending group sessions nested within a randomised controlled trial

**DOI:** 10.1186/1472-6874-9-25

**Published:** 2009-09-10

**Authors:** Frances Griffiths, Jo Pepper, Ellen C Jørstad-Stein, Jan Fereday Smith, Lesley Hill, Sarah (Sallie) E Lamb

**Affiliations:** 1 Health Sciences Research Unit, Warwick Medical School, University of Warwick, Coventry, CV4 7AL, UK; 2 Warwick Clinical Trials Unit, Warwick Medical School, University of Warwick, Coventry, CV4 7AL, UK; 3 Department of Psychology, Temple University, Weiss Hall, 1701 North 13th Street, Philadelphia, PA 19122, USA; 4 Physiotherapy Department, Warwick Hospital, Lakin Road, Warwick CV34 5BW, UK; 5 Physiotherapy Department, George Elliot Hospital, College Street, Nuneaton CV10 7DJ, UK

## Abstract

**Background:**

The aim was to explore the concerns and expectations of women invited to attend group physiotherapy sessions for the management of female urinary incontinence and whether the experience changed their views; and to gather recommendations from women attending group sessions on the design and delivery of these sessions

**Methods:**

An interview study nested within a randomised controlled trial in five British NHS physiotherapy departments, including 22 women who had expressed a preference for an individual physiotherapy session but were randomised to, and attended, group sessions.

**Results:**

Embarrassment was woven throughout women's accounts of experiencing urinary incontinence and seeking health care. Uncertainty about the nature of group sessions was a source of concern. Attending the first session was seen as a big hurdle by many women. However, a sense of relief was common once the session started, with most women describing some benefit from attendance. Recommendations for design and delivery of the sessions from women focused on reducing embarrassment and uncertainty prior to attendance.

**Conclusion:**

Taking account of women's embarrassment and providing detailed information about the content of group sessions will enable women to benefit from group physiotherapy sessions for the management of female urinary incontinence.

**Trial Registration:**

Trial registration number: ISRCTN 16772662

## Background

The provision of a group intervention by physiotherapists for female urinary incontinence is an attractive option for health care providers. The accompanying paper has demonstrated it to be of similar effectiveness to the same intervention on a one to one basis and group treatment should cost considerably less[1]. Earlier studies raised concerns about the uptake of group sessions for incontinence by women. An evaluation study[2] found relatively poor uptake of group sessions with only 41% attendance among women invited to a group session and only 35% of those invited completing the course. A pilot clinical trial of group versus individual sessions for incontinence found 35% of eligible patients declined to participate because of possible inclusion in the group arm, nearly half of whom expressed a preference for individual sessions and the rest declining due to inconvenient appointment times[3]. However other studies have not encountered this problem. One study showed that of the women attending group sessions for incontinence, most are satisfied with the group approach[4] and another that there was no difference in compliance between women randomised to group or individual sessions[5].

This paper reports the results of a qualitative study nested within a randomised controlled trial of a group versus individually delivered intervention for female urinary incontinence[1,6]. The group intervention is described below.

### Group intervention for female urinary incontinence

women receive an individual assessment prior to the groups, including a pelvic floor examination if indicated. Group met for three, one-hour long sessions over a three-week period. Group sizes were planned to be approximately 10 women. The physiotherapist leading the group delivered the following standardised information with Power-point slides.

#### First session

explanation of normal bladder function causes of stress incontinence, teaching and practice of pelvic floor exercises.

#### Second session

causes of urge incontinence, principles of bladder training, discussion and motivation, practice and progression of pelvic floor exercise, including exercises to target fast and slow twitch fibres, with a variation of starting positions.

#### Third session

a bladder quiz to re-enforce knowledge, avoidance of aggravating factors, repetition of pelvic floor exercises, discussion and motivation and safe lifting.

The women were given information booklets to take home with the same information

This paper reports women's concerns and expectations with regard to participation in such group settings and whether the experience of attending changed their views and makes recommendations for service providers for the design and delivery of group sessions in a way that maximises uptake.

## Methods

### Participants

The qualitative study recruited women in the following trial sub group:

Women who had agreed to participate in the clinical trial, expressed a preference for individual sessions, were randomised to group sessions and attended at least one session.

The clinical trial recruited adult women referred for stress, urge and mixed incontinence to one of five physiotherapy centres in the West Midlands of the UK in 2003/4. All women (n = 174) participating in the clinical trial were asked for consent to be approached for interview. Of the 111 women randomised to the group intervention arm, 7 withdrew from the trial and 11 received individual treatment as opposed to group treatment [1]. Of the 111 women randomised to group intervention 38 (34%) had expressed a baseline preference for individual treatment.

Women were recruited for interview until data saturation was reached. Twenty-three women were approached for interview of which 1 refused. Of the 22 women interviewed (please see Table 1), one had attended only one group session and two had attended two sessions. The 22 women were drawn from five of the centres participating in the clinical trial. Centre (A) contributed 13, (B) 4, (C) 2, (D) 2 and (E) 1. The recruitment ratio of trial: interview for each centre was (A) 81:13 (16%), (B) 32:4 (13%), (C) 32:2 (6%), (D) 31:2 (6%) and (E) 14:1 (7%). The variation was due to timing of the interview study in relation to trial recruitment.

**Table 1:** Characteristics of the 22 women interviewed collected at recruitment to the clinical trial using a structured questionnaire completed by recruiting physiotherapist

**Participant** **Identifier**	**Age** **years**	**Ethnicity**	**Employment** **Status**	**Did participant continue education after minimum school leaving age?**	**How would you describe the severity of your urinary problems or incontinence?**
A1	47	White	Working 20 h/week	No	Mild
A2	59	White	Unable to work due to illness/disability.	Yes	Moderate
A3	49	White	Working 20 h/week	No	Moderate
A4	57	White	Unable to work due to illness/disability	No	Mild
A5	30	White	On maternity leave	Yes	Moderate
A6	52	White	Unable to work due to illness/disability	Yes	Severe
A7	36	White	Working 15 h/week	Yes	Moderate
A8	59	White	Working 7 h/week	Yes	Moderate
A9	64	White	At home and not looking for paid employment	Yes	Severe
A10	43	White	Working 37 h/week	No	Mild
A11	51	White	Working 39 h/week	No	Moderate
A12	52	White	Working 35 h/week	Yes	Severe
A13	70	White	Working 6 h/week	No	Severe
C1	39	White	Working 24 h/week	Yes	Severe
C2	59	White	Working 37 h/week	No	Moderate
D1	63	White	Retired	Yes	Severe
D2	74	White EU	Retired	Yes	Severe
E1	66	White	Retired	No	Mild
B1	65	White	Retired	Yes	Mild
B2	36	White	Working 14 h/week	Yes	Moderate
B3	53	Indian	Working 40 h/week	No	Moderate
B4	50	White	Unable to work due to illness/disability	No	Severe

Note: all questions (except age) gave options for the response

Ethical approval was granted by Coventry Local Research Ethics Committee, Solihull Local Research Ethics Committee and Warwickshire Local Research Ethics Committee.

### Interview procedure

The semi-structured interviews aimed to hear the reflections of the women on the experience of attending group sessions for urinary incontinence and how it had affected them. The women were therefore contacted with regard to an interview approximately two weeks after completion of their treatment programme. Interviews were undertaken by a trained, non-clinical researcher. Of the 22 interviews 17, were completed within three months of completion and five within four months. The interviews lasted approximately one hour and all took place in the participants' home, except one that was conducted at the University.

An interview schedule was developed by the research team, including questions on expectations and concerns about group sessions before attending, the experience of being in the group session including how they felt, whether they talked to or compared themselves with others in the group, what they gained from the group session, how it could be improved and, whether their experience had changed their views about group sessions. The content and style of the interview questions was reviewed after the first few interviews. During the interview the women were encouraged explore issues in their own way but the interviewer tried to ensure all issues were covered. Participants were assured of confidentiality. Interviews were audio-recorded, transcribed and the transcription checked against the audio-recording for accuracy.

### Analysis

As this study was seeking women's views on very specific issues, thematic analysis was used [7] that followed the structure of the interview schedule. In addition, during their reading of the interview transcripts, the researchers looked for emerging themes not covered by the interview schedule but of importance to the women in relation to urinary incontinence. Three researchers (JP, FG and AD) initially read the same five transcripts, each drafting a list of themes. The researchers then met to discuss the themes and agree an initial coding scheme. One researcher (JP) then coded the interviews with a second researcher (FG) independently reviewing 1 in 4 interviews to check consistency of coding. New codes were added during analysis, where needed, although after 17 interviews no

new themes were identified. Coded sections from interviews were extracted and these were read again theme by theme, summarised descriptively and illustrated with quotations from the women. Disconfirming accounts were actively sought for each theme reported.

## Results

The women interviewed ranged in age from 30-74 years. The presence of self-rated severe symptoms was slightly more frequent in the interview group compared to the complete trial, and moderate symptoms were less frequent (see Table 1). The women interviewed were similar to those in the whole clinical trial in terms of age range, diversity of ethnicity, partnership status/living situation and employment status. Three women lived alone.

Embarrassment was woven throughout women's accounts of urinary incontinence and how they sought health care for the problem. We therefore start by exploring this emergent theme, examining how the women talked about their embarrassment. We then examine the data in relation to the issues specifically asked about in the interview, although embarrassment remains a strong theme throughout.

### Embarrassment

All the women interviewed talked about embarrassment related to urinary incontinence, although how they talked about it varied. Women talked about the embarrassment of having the problem: 'I do not want anyone else to know ... that I have got this problem' (C1), 'it's not the sort of thing you broadcast' (A7). Women had stories of situations where they had been very embarrassed by being 'wet' through their incontinence. These included when at work, particularly among male colleagues, and at social events. Several women talked about their fear of smelling, one woman commenting 'if I can smell then everyone else can smell me' (B4), and another talking about her experience of others:

waterworks is an embarrassing problem ... someone walks past you and they smell so bad, and you know they've got that problem... they're that used to the smell they don't even notice...ladies and men that smell a lot its horrendous (A11).

Others were less alarmed by their incontinence although still embarrassed. One woman described it as 'part of women's problems but still not something to talk about' (A1) and another said 'its not a dying problem ... its still personal' (A12).

All the women, except one, expressed feeling embarrassed talking to others about incontinence and discussed this in a variety of ways. Fourteen women spontaneously described urinary incontinence in terms of a taboo subject, something to be kept private, but there was diversity of how they coped with this prior to attending the treatment sessions. A few women admitted they had not mentioned the incontinence to anyone, even close family (E1, A13). Others did not mention whether they had discussed the issue before seeking health care. One woman felt able to talk to women friends who, like her, had recently had a baby (A5) and others had talked to family, friends and colleagues. Women also talked about their embarrassment on going to see a health professional about urinary incontinence. The one woman who said she was not embarrassed said 'We all pee and you know some of us have problems' (A6). This woman explained that her preference for one to one treatment was based on her experience of having psychiatric treatment where she built up a rapport with the therapist.

### Expectations and concerns about groups sessions before attending

All the women expressed uncertainty about what would happen in the group sessions and many were anxious about the sessions. For some the anxiety was mostly because of embarrassment and it becoming known that they suffered urinary incontinence 'you know they know what you are there for so it is not a private problem anymore' (C1). Several women admitted working out exit strategies if they found it too uncomfortable. Others talked about anxiety in relation to their uncertainty of what to expect 'I could not imagine how they would do it' (A8) and who would be there 'you never know who's going to be in there' (A5). Nearly half talked about specific expectations and concerns described below.

Several women described their vivid imaginings of the group sessions:

(I) had pre-conceived ideas of a workshop atmosphere, that it would be loads of little exercise mats on the floor, all lying down in positions in leotards (A5)

I thought there may be twenty five ...maybe they doing some treatment ... you have to take your clothes off or something (B3)

would we have to strip in front of one another? (A9)

One woman expected it to be like a support group where experiences were shared (A4) and another like an antenatal class, sitting in a circle chatting and less formal (B2). Several women expected the group to include women with the same problem, for example, women after childbirth (A5) or women with prolapse (B4). One woman expected the group to include women more able to express themselves than she felt she could (A8) and another felt concerned about being younger than others (B2). This sentiment was also expressed by another younger woman:

It would be nice to have people the same age in the same group I think that would have been better, you know to to have err a group of sort of like roughly the same age (C1)

Several women expected a much larger group (20-25 people was mentioned). Several women expressed concern about feeling 'judged', one describing it as being made to 'feel incompetent as well as incontinent' (A5).

### The experience of the group session

Attending the first session 'was a big hurdle' (A11) for some women as they had to build up their courage to go along. Sitting in the waiting room could be an ordeal:

They called out everybody for the knee class ... and for the hand class. I though what on earth are they going to shout out for (laughs) us? (A8)

You arrive there... there's lots of people arriving they give you the big blue book. after that you're holding your big blue book. ...so you can think oh she's going in for these sessions I think some people may have been a bit embarrassed about that (A10)

However, once the group session started, women described relief. This included relief that the session was a teaching session with no requirement for individual participation, relief on realising how common their problem was among women generally and relief from feeling isolated with their problem.

I most certainly did not feel rejected. by the group, but I didn't attempt to be part of a group it was like just going along to a lecture somewhere with people that you don't know at all (A8)

She didn't force you to say you know 'what's your problem? (A5)

She... explained that the same problem (urinary incontinence)... (affects) one in four (people) ... so we didn't feel that we were weird or different, you know, it was just a normal thing (A9)

You know there is someone around you that's got the same sort of problem. I mean it helps. There were others worse than me and I learnt a lot (A11)

A sense of relief was common to all the women in some form. However, the benefits the women described from attending the group session varied from greatly beneficial to no benefit. Some women described learning from other people's questions and experiences.

...we talked about triggers and things. One woman said that every time she gets off the bus she has to nip to the ladies... And somebody said 'oh yes that's me too' (B1)

Several women would have like more interaction in the group:

there was not a great deal of interaction.... I know people do not want to talk about their personal things but we met three times and I felt that it might have helped each other if we had been able to talk about our feelings (D1)

One described gaining information they could use themselves and pass on to friends and family. Another felt she had not receiving enough personal input from the physiotherapist and another had been put off seeking answers to her own questions at the end of the session because of the presence of others. One woman felt the group was irrelevant for her problem. Women also mentioned practical issues, such as transport difficulties, child care needs and the inflexibility of timing of sessions.

Most of the women had suggestions about how the group sessions could be improved. Many of these related to reducing anxiety in relation to embarrassment and uncertainty about what would happen in the group. In addition they had comments about the size of the group and how it was conducted. Some commented they liked the lack of interaction, others would have preferred more interaction in the group. For example:

if you only had a bit a time at the beginning or a bit of time at the end to talk with each other...you might find it easier, somebody else might have...you know a better way of doing it (C2)

The women were asked by the interviewer, now you have experienced the group, would you go to a group another time or recommend going to a group? An unequivocal 'yes' was given by all but three women. Women who would recommend the group commented on how the physiotherapist had put the group at their ease.

it was fine....she (physiotherapist) was very good, very nice and she did try to make bits of it funny. When we flew into the exercises ... (she made) most of the old women chuckle (A3).

One woman said she would recommend it, but:

I'd obviously want a one-to-one if I feel I've done my exercises like they were asking me to do and then perhaps you can get looked inside and someone say your muscles are this this and this (A12)

One of the three women who did not give an unequivocal 'yes' said, before deciding another time:

I'd like to know who's going to hold them ...what kind of qualifications this person had or experience ....what sort of help they can expect really whether it will be... medication or exercise ....also what people would be there I suppose how big a group it would be (D2)

Two women said they would not recommend group sessions. One said:

not for group therapy no, I don't think I got enough out of it (A2).

The other still held a preference for one-to-one sessions but was happy with the group she attended.

## Discussion

Women with urinary incontinence tend to be very embarrassed about the condition and this, along with a lack of information about what to expect from a group physiotherapy session, led them to prefer individual sessions for learning about bladder function and how to improve it. Most of the women were positive about the group sessions after experiencing them even though they did not all find them beneficial.

This study sought the views of women who had expressed a preference for one to one sessions and so are likely to represent the views of many women in the UK who find difficulty with accepting the idea of a group session. However, the study did not include women who refused to enter the trial, those who changed to individual sessions or those who withdrew during the trial. These women may have different views to the ones interviewed. Women were recruited until data saturation was achieved resulting in an uneven spread of women across recruitment centres due to the different pace of activity of the trial in the centres. However, no systematic variation by recruitment centre was detected during analysis of the interview content.

The theme of embarrassment was not specifically asked about in the interview but came across as the major issue for the women in this study. This was also an important theme in a UK study[8] which interviewed individuals identified with incontinence from a community survey, and showed embarrassment was one of a number of issues that prevented older people seeking treatment. In the study reported here, the women had already sought treatment, yet embarrassment still remained a major issue for them.

The interview data may be affected by the time between recruitment and interview. It may be difficult for women to recall how they felt up to four months earlier. They may also have had limited recall of how they felt before attending the sessions. The women may have wanted to avoid saying negative things about the service, so we need to treat with caution the positive responses to being asked about going to a group again, or recommending the group session. However, the women interviewed had many suggestions as to how to improve the group sessions, indicating a willingness to give criticism.

From our analysis of the interview data we have developed a list of recommendations for physiotherapy departments considering group sessions for female urinary incontinence (please see below). As these recommendations are drawn from the women interviewed they command our attention.

### Recommendations for group sessions run in physiotherapy departments for women with female urinary incontinence

Information sent to women prior to group sessions should include:

• details of what to do and what will happen on arrival at the physiotherapy department, including how they will be called into the session without embarrassment

• a description of the format of the sessions including length, expected size of the group, outline of the content of the sessions, the style of the sessions (e.g. a talk with illustrations on slides with participants seated throughout)

• introductory information about female urinary incontinence including how common it is

• opportunities for the women to ask questions both in the group and individually afterwards

• no requirement to share anything about themselves in the group

• no group activities such as floor exercises

• how women can choose times of attendance to their convenience

• opportunities for follow up with a physiotherapist after completion of the group sessions

In the waiting room:

• all staff need to be aware of the embarrassment felt by many women

• avoid handing out information about incontinence in the waiting room

• call women in by their name

The session:

• provide sessions at a variety of times of day so women can choose

• consider providing groups for different age ranges or where this is not feasible in the group session acknowledge the different age groups that are present and how the approach to the problem is similar for all ages

• at the start explain format of the sessions

• remind women about opportunities for asking questions in the group and after the group

• after the group session allow time and space for individuals to ask questions without being over heard

Overall, the study indicates that most women were happy with being in a group for receiving the intervention. It was not knowing what this would be that caused apprehension and this was exacerbated by their embarrassment. Some women felt they needed individual time with a physiotherapist for clarifying their individual needs and follow up, but this was identified by the women as different from the teaching about bladder function delivered in the group sessions. Women who have asked for help with their urinary incontinence are embarrassed about their incontinence. Taking account of this embarrassment is a key factor for the success of group sessions, and teaching about bladder function for those with female urinary incontinence.

## Conclusion

Taking account of women's embarrassment and providing detailed information about the content of group sessions will enable women to benefit from group physiotherapy sessions for the management of female urinary incontinence.

## Competing interests

The authors declare that they have no competing interests.

## Authors' contributions

JF-S and LH had the original concept for the study. SELgained funding, and oversaw the study management. JP undertook the qualitative interviews under the guidance of FG. FG, JP and ECJ-S analysed the data. JG drafted the manuscript, and all authors have commented on the submission. All authors have read and approved the final manuscript.

## Pre-publication history

The pre-publication history for this paper can be accessed here:


